# Influence of the spaceflight environment on macrophage lineages

**DOI:** 10.1038/s41526-023-00293-0

**Published:** 2024-06-11

**Authors:** Rocky An, Virginia Katherine Blackwell, Bijan Harandi, Alicia C. Gibbons, Olivia Siu, Iris Irby, Amy Rees, Nadjet Cornejal, Kristina M. Sattler, Tao Sheng, Nicholas C. Syracuse, David Loftus, Sergio R. Santa Maria, Egle Cekanaviciute, Sigrid S. Reinsch, Hami E. Ray, Amber M. Paul

**Affiliations:** 1 NASA Ames Research Center, Space Life Sciences Training Program, Moffett Field, CA 94035 USA; 2 Cornell University, Department of Biological and Environmental Engineering and Sibley School of Mechanical and Aerospace Engineering, Ithaca, NY 14853 USA; 3 Massachusetts Institute of Technology, Department of Biology, Cambridge, MA 02139 USA; 4 Tufts University, Department of Chemistry, Medford, MA 02155 USA; 5 University of California San Diego, Department of Cellular and Molecular Medicine, La Jolla, CA 92093 USA; 6 Embry-Riddle Aeronautical University, Department of Human Factors and Behavioral Neurobiology, Daytona Beach, FL 32114 USA; 7 Georgia Institute of Technology, Atlanta, GA 30332 USA; 8 Medical University of South Carolina, Charleston, SC 29425 USA; 9 Brooklyn College, Department of Natural and Behavioral Sciences, Brooklyn, NY 11210 USA; 10 Ohio State University, Department of Physiology and Cell Biology, Columbus, OH 43210 USA; 11 University of Pittsburgh, Department of Computer Science, Pittsburgh, PA 15260 USA; 12 North Carolina State University, Department of Molecular and Structural Biochemistry and Department of Biological Sciences, Raleigh, NC 27695 USA; 13 NASA Ames Research Center, Space Biosciences Division, Moffett Field, CA 94035 USA; 14 ASRC Federal, Inc, Beltsville, MD 20705 USA; 15 Blue Marble Space Institute of Science, Seattle, WA 98104 USA

**Keywords:** Biomarkers, Immunology

## Abstract

Spaceflight and terrestrial spaceflight analogs can alter immune phenotypes. Macrophages are important immune cells that bridge the innate and adaptive immune systems and participate in immunoregulatory processes of homeostasis. Furthermore, macrophages are critically involved in initiating immunity, defending against injury and infection, and are also involved in immune resolution and wound healing. Heterogeneous populations of macrophage-type cells reside in many tissues and cause a variety of tissue-specific effects through direct or indirect interactions with other physiological systems, including the nervous and endocrine systems. It is vital to understand how macrophages respond to the unique environment of space to safeguard crew members with appropriate countermeasures for future missions in low Earth orbit and beyond. This review highlights current literature on macrophage responses to spaceflight and spaceflight analogs.

## Introduction

Space exploration and habitation will expose crew members to unique risk factors, including cosmic radiation, altered gravity forces, social isolation, and enclosed/hostile environments. In addition, these stressors will be experienced at a substantial distance from Earth, where emergency medical intervention will be limited. Multiple studies on crew health and model organisms have determined spaceflight-associated risks can impact nervous, musculoskeletal, and cardiovascular systems (reviewed in^1^). Tissue-resident macrophages, circulating blood monocytes, and lymphatic system responses to spaceflight-associated risk factors can influence physiological outcomes in biological systems. Studies performed on space exploration vehicles and on the International Space Station (ISS) have yielded varied findings on immunological patterns, which may be in part due to limitations to onboard sampling and experimental procedures. In general, crew members who have flown in space display altered leukocyte counts and function, as well as chronic low-grade inflammation^2–9^. Chronic inflammation experienced in-flight may be in response to oxidative damage^10^ (reviewed in^11^), which may also contribute to accelerated aging described post-flight^12,13^. However, due the dynamic nature of the immune system, contributions of circadian cycling, epigenetics, and differential response kinetics are only now beginning to be evaluated. Thus, understanding the impact of spaceflight-associated risks on macrophage phenotypes and functions is paramount for astronaut health monitoring and mitigation programs.

Macrophages are key elements of the innate immune response and play an important role in antigen removal via phagocytosis. They also direct the adaptive immune response through antigen presentation and lymphocyte immunological synapse formation. In addition, many non-genetic, multisystem terrestrial diseases are caused by aberrant inflammation, which is in part regulated by macrophages (reviewed in^14,15^).

There is a significant knowledge gap in the characterization of macrophage heterogeneity involved in spaceflight-associated dysfunctions, in addition to identifying homeostatic resolution processes. Consequently, teasing apart cell type-specific responses may help characterize distinct immunological processes observed in the spaceflight environment.

## Macrophage immunobiology in spaceflight

Immune cell phenotypes and functions are altered in spaceflight, with known consequences for monocytes and macrophages (Table 1). For instance, elevated monocyte counts were described in crew at 1-day post-flight (>142-day mission)^8^. In line with this, ISS crew monocyte counts are not significantly altered at early- (14-days), mid- (2–4-months) and late-(6-months) timepoints in-flight, however, are slightly increased immediately upon return to 1 g and at 30-days post-flight^7^. Collectively, these findings indicate elevated monocyte counts during post-flight may be a factor of immune resolution and recovery. In rodents, on the other hand, absolute monocyte counts isolated from splenocytes were reduced immediately post-flight^16^. Similarly, other studies in rats report reduced post-flight monocyte counts upon return from a 14-day mission^17^.

**Table 1:** Spaceflight-induced Macrophage Lineage Phenotypes.

Spaceflight-Induced Phenotype	Macrophage Lineage involvement	In-flight or Post-flight	Mission Duration	Time of collection and observation	Organism	Reference
Increased monocyte counts	Direct	Post-flight	6-month	R + 0 and 30-days	Human	^7^
Increased monocyte counts	Direct	Post-flight	142–181 days	R + 1	Human	^8^
Reduced monocyte counts	Direct	Post-flight	13-days	R + 0	Mouse	^16^
Reduced monocyte counts	Direct	Post-flight	14-days	R + 0	Rat	^17^
Reduced phagocytic function	Direct	Post-flight	8–10 days	R + 0	Human	^28^
Reduced phagocytic function	Direct	Post-flight	125–195 days	R + 0	Human	^28^
Reduced phagocytic function	Direct	Post-flight	5–11 days	R + 0 and R + 3	Human	^29^
Reduced TNF-α, IL-6, and IL-10 cytokine expression	Direct	Post-flight	13–16-day	Post-flight (R + 0) post-LPS stimulation	Human	^32^
Elevated CCL2, IL-10, CRP, IL-6, and IL-1RA	Indirect	Post-flight	340-days	R + 0	Human^a^	^22^
Impaired antigen-specific tolerance	Indirect	Post-flight	15-day	R + 0 and R + 3	Mouse	^35^
Elevated TNF-α, IL-17F, and IL-6	Indirect	In-flight	334-days	Day 14, 74, 122, 181, 237, 300, and 334	Human^a^	^2^
Elevated IL-1RA, IL1-α, IL-1β	Indirect	In-flight	4–6 months	Day 15	Human	^3^
Elevated CXCL-8 (IL-8) and CXCL-5	Indirect	In-flight	6-months	Day 15, 60, & 180	Human	^5^
Elevated IL-12p40	Indirect	In-flight	136–290-days	Day 15, 30, 60, 120, & 180	Human	^6^
Elevated TNF-α and IL-1β	Direct	In-flight	6-day mission	12- and 24-hours post-LPS stimulation	Mouse	^23^
Reduced macrophage differentiation from HPC	Direct	In-flight	12-days	Day 1–12; every 24-hour image capture	Mouse	^24^

^a^n = 1 biological sample; “HPC” denote “Hematopoietic Progenitor Cells”; “R + 0” denotes “sampling within 24 h at post-flight landing”; “R + 1” denotes “sampling one-day post-landing”; “R + 3” denotes “sampling three days post-landing”.

Immune discrepancies between rodent and human mammalian systems are not entirely uncommon and may be due to multiple variables, including the strain of experimental mouse, multi-physiological system influence, age, sex, duration of spaceflight exposure, environmental microbial influence, methods or markers of analysis, and/or landing experiences. Moreover, rodent quadrupedal models do not recapitulate the bipedal human with an upright, stacked vertebrae spinal column. Therefore, simulated gravity models impact rodents and humans differently (reviewed in^18^), and may have important implications for monocyte (and other immune cell) functions. The majority of simulated microgravity studies in rodents utilize the hindlimb unloading (HU) model, which lifts rodent hindquarters to cause a cephalad fluid shift similar to what humans experience in spaceflight (reviewed in^19^). Immunological consequences observed following HU and spaceflight are somewhat similar, such as thymus involution and induced cytokine-type phenotypes (reviewed in^20^). Additionally, leukocyte differentials are also generally similar between HU rodent models and humans post-flight, including elevated production of inflammatory mediators (reviewed in^21^). However, careful consideration of spaceflight analog details and a thorough elucidation of all descriptive metadata from spaceflight missions are important for immunological interpretation.

**Fig. 1:**
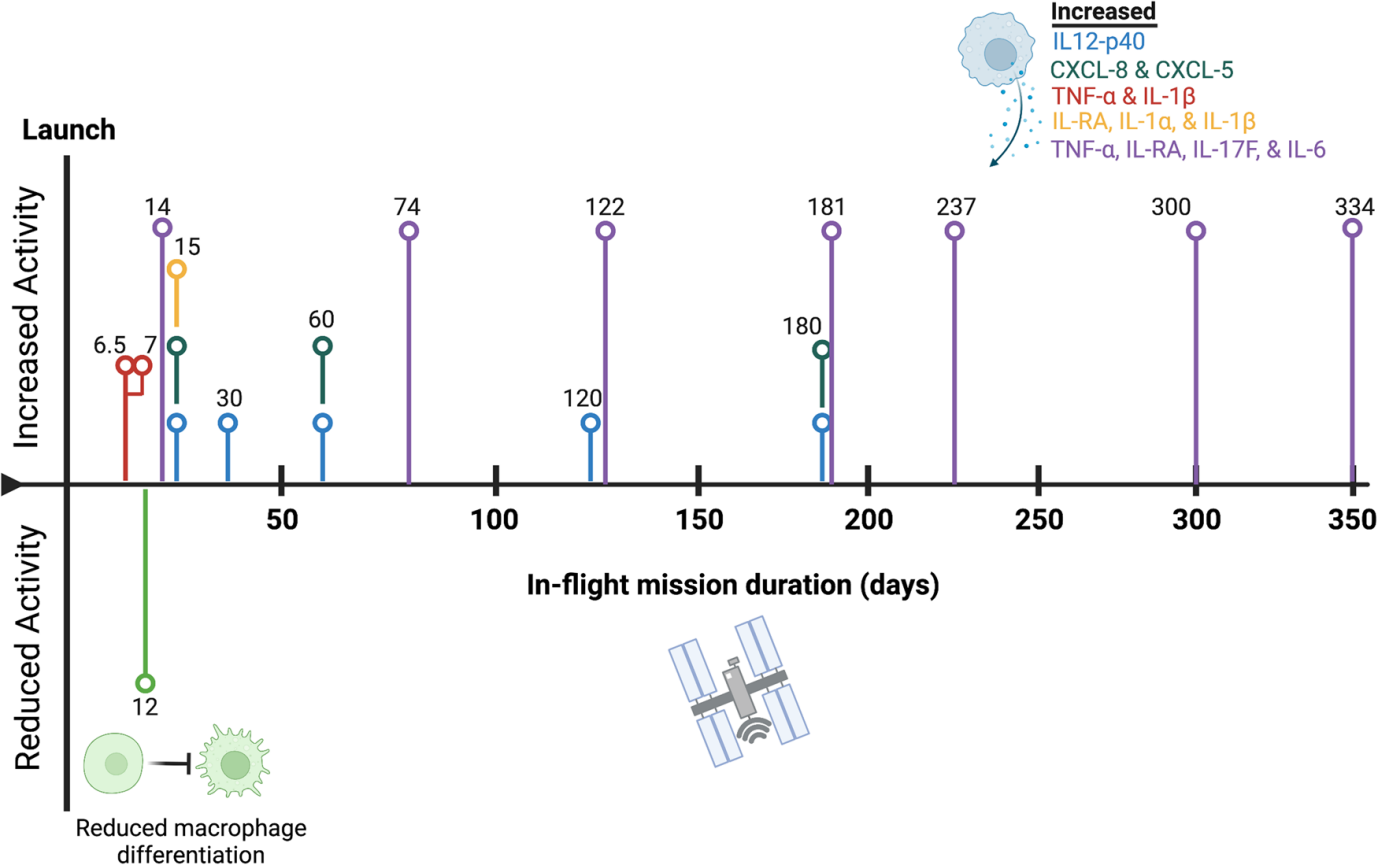
In-Flight Macrophage Lineage Phenotypes. Representative timeline of the observable phenotypes produced in-flight from Table 1. Each line/color represents a mission experiment with the in-flight days listed above/below vertical nodes. Collective observable phenotypes display increased (above centerline) or decreased (below centerline) activity are depicted visually, including increased production of IL12-p40, CXCL-8, CXCL-5, TNF-α, IL-1β, IL-RA, IL-1α, IL-17F, and IL-6 and decreased macrophage differentiation processes from hematopoietic progenitor cells (HPC). Created with BioRender.com.

In-flight changes of known macrophage lineage mediators have also been reported in crew members. For example, IL12-p40^6^, CXCL-8/IL8, and CXCL-5^5^ cytokines are elevated throughout multiple timepoints, indicating the potential for some degree of macrophage activation under microgravity conditions. In addition, multipaneled measurements of mediators indirectly involved in macrophage activation are elevated in-flight, including IL-1α, IL-1β, and IL-1RA^3^, and TNF-α, IL-17F, and IL-6^2^. In line with this, the production of immune mediators CCL-2, IL-10, CRP, IL-6, and IL-1RA are substantially elevated immediately at landing (340-day mission) in the NASA Twins study (n = 1) that were quickly reversed in post-flight recovery^22^. Induction of TNF-α and IL-1β were also described following in-flight stimulation of the murine bone marrow-derived cell line, B6MP102 cells, with lipopolysaccharide (LPS, 12- and 24-hours) on-board STS-37 (6-day mission duration)^23^. In addition, although macrophage polarization profiles are beginning to be identified in simulated microgravity models and spaceflight^24,25^ (reviewed in^26,27^), more studies on these unique phenotypes may better assist with unraveling functional consequences of macrophage alterations in spaceflight (Fig. 1). Recently, Lv et al. discussed a negative role for microgravity on hematopoietic progenitor cells (HPC) differentiation and polarization processes, including non-polarized (M0), pro-inflammatory (M1), and anti-inflammatory (M2) subtypes. Under microgravity and simulated microgravity conditions, differentiation of HPC into macrophages and the polarization of macrophages into M1 or M2 types were mutually impaired^24,25^ (reviewed in^26,27^). Thus, spaceflight risk factors may influence macrophage differentiation/polarization processes in flight.

In a post-flight study, human monocytes displayed reduced phagocytic activity immediately following short- (8–10 days) and long- (125–195 days) duration spaceflight missions^28^. Another study found that monocyte phagocytic function was depressed immediately upon return to Earth (within 3 h) and at day 3 post-flight^29^. Murine splenocyte phagocytosis was also impaired immediately post-flight (within 5 h) after a 13-day spaceflight mission^16^. The inability to remove cellular debris, apoptotic cells, or pathogens can impede tissue regeneration, nutrient recycling, and can cause tissue damage, which is also a notable dysfunction in the elderly (reviewed in^30^). Concordantly, astronauts display accelerated aging phenotypes in peripheral blood mononuclear cells (PBMC) post-one-year missions via telomere shortening mechanics^2,12^, suggesting post-flight macrophages may also have an accelerated age phenotype, that is possibly inflammatory^12^ (reviewed in^31^). However, previous work described reduced monocyte expression levels of pro-inflammatory TNF-α, IL-6, and immunoregulatory IL-10 upon LPS ex vivo stimulation, post-short duration spaceflight shuttle missions (13–16 day missions, STS-124, STS-125, and STS-126)^32^. Differences in study outcomes may be a due to variables including duration in spaceflight and types of mission exposures (i.e., shuttle versus ISS). Additionally, while recovery to baseline macrophage function may be achieved post-flight on Earth, the time frame to recovery is an important consideration for future exploration missions on lunar and Martian surfaces.

**Fig. 2:**
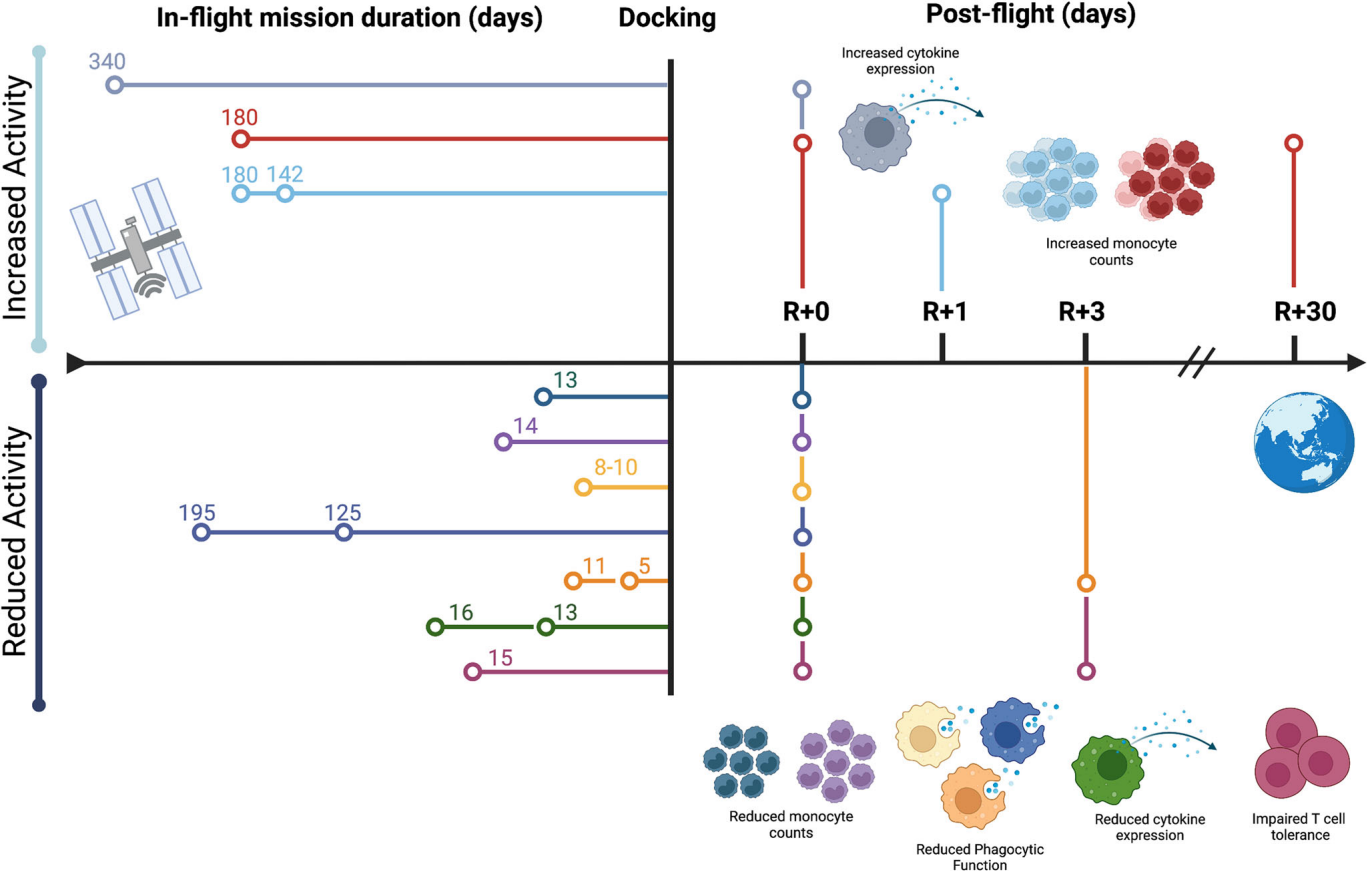
Post-Flight Macrophage Lineage Phenotypes. Representative timeline of the observable phenotypes produced post-flight analysis from Table 1. Each line/color represents a mission experiment with the in-flight days listed above horizontal nodes, and post-flight collection time points vertical lines. Collective observable phenotypes that display increased (above centerline) or decreased (below centerline) activity are depicted visually, including monocytic count, phagocytic function, cytokine production, and T cell tolerance outcomes. Each phenotype is color-coordinated with the experimental timeline of missions and collections. Created with BioRender.com.

Macrophages are also critically involved in directing type-specific adaptive immunity via antigen presentation and the production of lymphocyte differentiation cytokines (reviewed in^33^). Spaceflight can alter adaptive immunity through impaired T cell function^7^ and impaired lymphocyte maturation (reviewed in^34^), both of which can cause immune deficiency. As macrophages play an important role in antigen presentation and can shape lymphocyte effector cell phenotypes, we hypothesize that altered macrophages may significantly alter adaptive immune phenotypes observed during spaceflight. However, due to the complexity of performing in-flight studies to characterize immunological synapses or macrophage-lymphocyte communication processes, these studies have been lacking to date. Nonetheless, one study has characterized the potential for peripheral immune tolerance processes being disrupted in space-flown mice (15-day mission), whereby antigen-specific tolerance, mediated by antigen-presenting cells, including macrophages, was compromised and elevated inflammation was recorded post-flight^35^. This study highlights the potential for fundamental antigen presentation/processing mechanisms being compromised in-flight. Thus, deficits in antigen processing and presentation can influence inappropriate effector lymphocyte establishment, which may be the basis for reported adaptive immune dysfunctions in flight^7^, although additional studies are required (Fig. 2).

Most studies that characterize macrophage lineage biology and function in response to the spaceflight environment are limited to post-flight outcomes, although some have characterized in-flight cell counts and cytokine/chemokine profiles. As such, additional experimental evidence is necessary to fully characterize unique phenotypes of monocytes and macrophages in spaceflight, including functional outcomes on lymphocyte populations.

## Tissue-specific macrophage lineages in spaceflight

As described above, macrophages can undergo alterations in the spaceflight environment, which may in part contribute to spaceflight-induced conditions. Specialized tissue-specific macrophage lineages in the spaceflight environment are less characterized, such as microglia of the CNS or bone osteoclasts (reviewed in^36^). Yet, there are some intriguing spaceflight environmental consequences on these specialized tissue macrophage lineages that have been identified, displaying similarities to known terrestrial disorders. For instance, the spaceflight environment (12-day mission murine OSTEO payload) has known effects on osteoclasts resulting in osteoclastogenesis and increased bone resorption^37^, which may play an important role in exacerbating bone loss in spaceflight, resembling osteoporosis (reviewed in^38,39^). Furthermore, microglia are brain-resident macrophages that are responsible for the maintenance of brain homeostasis by surveying the microenvironment in a resting state. However, when activated, microglia can play a contributing role in neuroinflammation causing bystander tissue damage if unresolved (reviewed in^40^). Similar to features resembling Alzheimer’s disease^41^, microglia can be activated by space-relevant galactic cosmic rays (GCR) doses both in vitro and in vivo models of cosmic radiation (reviewed in^42,43^), leading to cognitive deficits and neuroinflammation in mice^44^. Interestingly, the removal of microglial populations through cellular depletion in mice can prevent sex-specific GCR effects such as learning deficits and phagocytic activation^45^, and indicates a potential target for neuroinflammatory regulation in spaceflight-associated radiation models (reviewed in^46^). Table 2 summarizes some of the similarities identified between various specialized macrophage lineages in spaceflight and associated terrestrial disorders.

**Table 2:** Specialized tissue-specific macrophage lineages in spaceflight.

Macrophage Location & Lineage	Spaceflight or analog consequences	Related terrestrial disorders	Reference
Bone Osteoclast	Increased differentiation, bone resorption, and loss of bone calcium	Osteoporosis	^37,38,52^
Central Nervous System Microglia	Cognitive deficits and neuroinflammation	Neuroinflammation and Alzheimer’s Disease	^41–46^
Small & large Intestine lamina propria mucosa macrophages	Altered gut microbiome	Gut dysbiosis	^90^
Liver Kupffer cells	Reduced populations in liver	Liver disease	^48,49^
Pancreatic-resident Macrophage	Unknown function	Metabolic disorders and insulin resistance	^50^
Kidney-resident Macrophage	Unknown function	Kidney stone formation	^51,52^
Adrenal-resident Macrophage	Unknown function	Metabolic disorders, endocrine hormone release, and circadian cycle homeostasis	^50,82,83,89^

There are several research gaps characterizing macrophage lineages in the spaceflight environment and are of interest for future spaceflight studies (Table 2). For example, Kupffer cells of the liver are part of the hepatic macrophage system and play an important role in liver immunological tolerance, producing IL-10 and high PD-L1 expression (reviewed in^47^). Reduced rat Kupffer cell populations have been previously reported in hepatic tissues post-spaceflight (14-day mission)^48^, suggesting possible disruption in the maintenance of liver homeostasis and may play a significant role in immunological resolution during inflammatory-induced conditions in spaceflight (reviewed in^49^), however, more studies are necessary. Similarly, macrophages within other less characterized tissues, such as the pancreas, kidneys, and adrenals also may play important roles, due to parallel terrestrial disorder overlaps with spaceflight-associated conditions of insulin resistance, metabolic disorders (reviewed in^50^) and renal stone formation risk^51,52^. However more studies elucidating the role of specialized tissue-resident macrophages lineages are required.

## Influence of microgravity on macrophage lineages

Ground-based, terrestrial analogs of microgravity that assess cells in culture include rotating wall vessel (RWV) bioreactors or rotary cell culture systems (RCCS), and clinostats (2D/3D) or random positioning machines. Other possible analogs that are less utilized include, drop towers and parabolic flight (reviewed in^53^). As described above, HU and partial weight-bearing suspension are standard rodent models used to simulate microgravity, while head-down tilt bead rest and wet/dry immersions are used in humans (reviewed in^53,54^). However, analogs can produce disparate responses to true microgravity experienced in spaceflight on the ISS, therefore highlighting the importance of utilizing the ISS for future experiments in this field. For example, 2D clinostats can possibly induce spurious fluid motion and shear stress^55^, which can have negative immune cell consequences. Further, unless otherwise designed, ground-based microgravity analogs do not include other risk factors that are experienced in the spaceflight environment, such as ionizing radiation and elevated carbon dioxide levels, for example^1^. Therefore, accurately modeling immune function in simulated microgravity analogs that is similar to spaceflight requires careful attention to experimental design.

Considering mechanical and intercellular communication between heterogeneous populations of cells and the hypothesis that the cellular cytoskeleton has built-in mechanisms for sensing mechanical stress (reviewed in^56,57^), it is possible that macrophages may also be sensitive to mechanical stress. Mechanical stress includes stretch and compressive forces, and hydrostatic shear pressures that are experienced within multiple tissue types (reviewed in^58^), where macrophages reside. Mechanical unloading, or decreased mechanical stress on cells and tissues can simulate microgravity (reviewed in^59^). At the cellular level, mechanical unloading causes pathological phenotypes via altered cellular mechanotransduction pathways, which may reflect cellular changes in astronauts (reviewed in^57^). Although mechanosensitivity of macrophages in vivo has not been characterized in spaceflight, several human spaceflight missions report altered cytokine expression profiles^2,4,8,28,32,60^, which may be a response of altered mechanotransductive signaling cascades in macrophages (reviewed in^26,57^). These findings underscore complex and dynamic responses in the spaceflight environment and highlight a gap in knowledge that requires further studies.

Microgravity analogs can cause actin reorganization and changes in cytoskeletal and nuclear morphology^61^, which may impact transcriptional or replicative programs. In addition, the plasma membrane physically couples to cytoskeletal anchoring proteins and contains Piezo mechanosensitive channels that transduce mechanical stimuli into electrical signals (reviewed in^62^), which may be an important target for future microgravity research. In line with this, the FAK-ERK1/2 signaling pathway involved in actin polymerization and multiple other signal transduction cascades, is downregulated in HPC concomitantly differentiating into macrophages (12-day differentiation process) both in simulated microgravity conditions (12-days) and spaceflight (12-day mission)^24^, indicating aberrant cell signaling, impaired macrophage maturation, and cytoskeletal disturbances are influenced by the microgravity environment. In line with this, signaling pathways such as STAT3, P38, JNK, FAK-ERK, Rho/ROCK, AKT, CREB, NF-κB, RAC-WAVE-Arp2/3 (reviewed in^63,64^) and MRTF-A/SRF (reviewed in^64,65^), may all be engaged in microgravity, which also requires further studies.

Monocyte/macrophage migratory behavior is also altered in both simulated microgravity and spaceflight conditions. For example, integrin intracellular adhesion molecule-1 (ICAM-1), which regulates cellular migration and extravasation, is induced in differentiated U937 macrophage-like cells and BV-2 microglia cells, following rotating wall vessel and parabolic flight (reviewed in^66^). ICAM-1 is also elevated at 120 h of spaceflight^67^, which may result in enhanced extravasation processes and immune activity. Thus, microgravity poses a unique and varied environmental change that may require quantitative, comparative, and multifactorial approaches to tease out pathological macrophage mechanisms at the cellular, tissue, and physiological scales.

## Cosmic ionizing radiation and macrophage destabilization

Exposure to photon and particle cosmic radiation, including gamma, X-ray, solar particle events (SPE) and GCR, may further influence macrophage dysfunction during deep space missions. The complex space radiation environment is nearly impossible to simulate on Earth, thus research on space-related ionizing radiation has historically utilized gamma rays and single ion particles^68^. With the introduction of the newly designed simulated GCR (33-ion sequential beam and simplified 5-ion beam) and SPE dosing schemes (including protracted and acute exposures) developed by the NASA Space Radiation Laboratory (NSRL) at Brookhaven National Laboratory, more opportunities are available to simulate some aspects of the deep space radiation environment^69,70^.

GCRs are composed of high-energy charged particles, primarily protons, helium ions, and high mass/high energy (HZE) particles^68,70^, all of which can have detrimental effects on biology (reviewed in^71^). Characterization of monocyte and macrophage responses to GCRs or their components currently remains inadequately defined. On the one hand, via the metric of apoptosis, macrophages are more radioresistant compared to monocytes, while the lymphoid lineage is even more radiosensitive^72^. Mice exposed to simulated 5-ion simplified GCR (0.5 Gy) and SPE (1 Gy) irradiation with and without HU displayed no differences in monocyte counts, although reduction in lymphocytes were observed 24-hours post-exposure^3^. Other studies have reported elevated blood monocyte counts seven-days post-GCR irradiation at comparable doses (0.5 Gy), which positively correlated with impaired spatial learning at five months post-exposure in mice^45^. This suggests that radiation-mediated damage during myelopoiesis may impact cognitive performance post-irradiation. This effect might be primarily mediated by brain-resident macrophages (microglia), as depleting microglia also improved cognitive outcomes after irradiation^45^(reviewed in^73,74^).

Although not a comprehensive analog to space radiation, cancer radiotherapy studies have provided some insight into immune phenotypes following ionizing radiation. Indeed, macrophages play an important role in both regulation and resolution of inflammation, for instance certain polarized types of macrophages are prominent in the progression of cancer, while radiotherapy may promote antitumor phenotypes (reviewed in^75^). Many characteristic inflammatory profiles produced by macrophages depend on cellular abundance and response to danger-associated molecular patterns (DAMPs), produced from radiation-induced DNA damage^76^. Thus, in spaceflight, damaged DNA may trigger DAMP receptor pathways inducing inflammatory mediator production^2–4,8^.

Notably, space mission-relevant doses of X-ray irradiation (0.1, 0.5, 1, and 2 Gy) did not alter macrophage phenotypes^77^. Interestingly, dose-dependence did alter macrophage phenotypes when cells were cultured in the presence of radiation-conditioned fibroblast supernatant, suggesting physiological mediators, received from other cells were necessary to generate unique cellular phenotypes in response to radiation^77^. Thus, the complex nature of the space radiation environment and its energies, doses, and dose rates make assessing macrophages function a difficult task. Future studies on this topic will require better characterization of macrophage polarity and responses following acute and protracted doses, combination spaceflight exposures, and longitudinal immune sampling.

## Isolation, confinement, and extreme environmental stressors

Isolation, confinement, and extreme (ICE) environments also pose unique risks to crewmembers on exploration missions^78^. Operational on-board stressors that fall into this category include circadian misalignment, social isolation, and closed/hostile environments, all of which may impact macrophage biology. Spaceflight and ground analogs indicate that ICE may disrupt circadian rhythms critical to immune functions (reviewed in^79,80^). More specifically, ICE may impact circadian misalignment by manifesting disruptions in monocyte and macrophage molecular clocks (reviewed in^81^). Indeed, light (in the form of visible sunlight) is a dominant environmental cue for many biological clock physiological processes, such as sleep/wake, endocrine hormone release, metabolic processes, and temperature regulation (reviewed in^82^). Circadian misalignment, as a result from irregular light cues in spaceflight, can cause deviations from normal cycling and may have profound impacts on multiple physiological systems^83^. In line with this, circadian misalignment may innately impact immune function. Indeed, there have been reports describing cell-autonomous clock gene expressions in rat natural killer cells^84^ and mouse peritoneal macrophages^85^. Notably, in the absence of the core clock component protein cryptochrome (CRY), elevated proinflammatory cytokine expression mediated through NF-κB activation has been reported^86^. Further as mentioned above, neuroendocrine hormone release is regulated by the circadian system. For example, glucocorticoid release peaks in the early morning in response to light (diurnal), which integrates with cyclic systemic immunity (reviewed in^87^), suggesting neuroendocrine system crosstalk with immunity. Indeed, glucocorticoids can also directly impact macrophage polarization phenotypes, indirectly linking immune regulation to circadian cycling^88^. Therefore, modulation of glucocorticoid production that aligns with circadian cycle regulation in astronauts on deep space missions should be considered in countermeasure designs. Furthermore, diurnal sample collection metadata should also be evaluated during immune response analysis and considered for future experimental design.

Social isolation in mice in combination with HU caused population shifting in leukocytes, including neutrophils and lymphocytes, while monocyte populations were unaltered^89^. These studies further support crosstalk with the neuroendocrine hypothalamus-pituitary-adrenal (HPA) axis is involved in immune regulation. In addition, immune differentials reported in the Mars500 isolation project in humans indicate the effects of extreme isolation on gut microbiome maintenance and immunity, a site populated by macrophages^90^. In line with this, multiple other isolation analogs, including NEEMO, Antarctica, and MOON-2015 have assessed the influence of isolation on macrophage lineage phenotypes (primarily monocytes), collectively indicating differential outcomes are observed during- and post-study collections (reviewed in^91^). Differential outcomes may be due to the unique microbial environment present in each analog, further adding to the complexity of macrophage phenotype characterizations. Thus, an important future research direction would be to determine the extent of spaceflight-caused macrophage dysfunction and the mechanistic underpinnings involved in macrophage function as a result of ICE-related operational stressors, along with considering environmental microbial influence.

## Hypercapnia and macrophage function

Increased partial pressure of CO_2_ aboard the ISS might also contribute to spaceflight-induced immune dysfunction. It is well-accepted that elevated levels of environmental CO_2_ can cause hypercapnia (elevated blood/tissue CO_2_ levels). Elevated levels of CO_2_ impair macrophage ability to defend against foreign invaders by inhibiting the production of cytokines critical for antimicrobial host defense, such as TNF and IL-6^92^. Hypercapnia also downregulates genes associated with innate immunity, antiviral response, and cytokine signaling in both human and mouse macrophages^93^, while on the cellular level, hypercapnia has been shown to cause macrophage apoptosis and decreased phagocytic activity^92,94^. For example, hypercapnia can increase influenza A virus replication and inhibit antiviral gene and protein expression in mouse macrophages, which is dependent on the activity of Akt^95^. Indeed, Akt isoforms can modulate Akt activity levels in macrophages and their polarization phenotypes^96^; therefore, Akt may be a potential therapeutic target to enhance macrophage host defense. Collectively, these findings suggest deficits in macrophage immunity can be caused by hypercapnic conditions. This emphasizes a critical risk to consider for long-duration ( > 1 year) exploration missions, where exposures to elevated CO_2_ levels may be experienced for prolonged periods of time.

## Macrophages and future lunar mission considerations

Lunar dust poses a serious challenge for exploration missions to the moon, but the limited availability of authentic lunar dust samples makes it difficult to conduct biological experiments on Earth. Studies of Apollo 14 lunar dust exposure in rats demonstrated lung toxicity when inhaled (reviewed in^97^). Further, an in vitro study assessed the behavior of a transformed macrophage cell line in the presence of SiO_2_ and Al_2_O_3_ (analogs of lunar dust). In the presence of these mineral particles, phagocytosis was impaired^98^ and inducible nitric oxide synthase (iNOS) was increased^99^. In addition, mouse alveolar macrophage counts decrease following lunar dust exposure, along with neutrophil aggregation^100^, suggesting increased cell death pathways and inflammation in the lung microenvironment may be involved in the pathophysiology of lunar dust exposure. Since macrophages play a major role in phagocytosis and defense against toxins, further studies on macrophage function following lunar dust and other celestial dust exposures are essential for mitigation agendas on future lunar missions.

## Future outlook and summary

A growing body of experimental evidence, reviewed in this monograph, indicates that monocytes and macrophages are altered by the spaceflight environment. These findings have implications for a wide range of physiological processes, including innate immunity, acquired immunity, host defense, and tissue remodeling. Aside from spaceflight, the studies described in this review involve the examination of a single aspect of the space environment (such as weightlessness, space radiation or elevated CO_2_ levels). Future experiments involving combinations of spaceflight stressors, such as elevated CO_2_ levels combined with simulated space radiation and gravitational changes, would enable a more comprehensive understanding of the effects of the spaceflight environment on macrophage function. Further investigations of cell- and tissue-specific macrophage responses and phenotypes are also necessary to assess tissue-specific pathologies that connect cellular studies to human disease processes on long-duration missions in deep space. Considering the immune system “computes” the state of health throughout the body (reviewed in^96^) additional assessment of other immune cell types in the hematopoietic tree is also necessary. In brief, fundamental studies on macrophages in space have begun to lay the groundwork for the development of targeted countermeasures that optimize macrophage function and are needed to address clinical challenges that arise as space exploration moves beyond low Earth orbit.

### Reporting summary

Further information on research design is available in the Nature Research Reporting Summary linked to this article.

### Supplementary information

Reporting Summary
